# Daily stair climbing is associated with decreased risk for the metabolic syndrome

**DOI:** 10.1186/s12889-021-10965-9

**Published:** 2021-05-14

**Authors:** Anna C. Whittaker, Frank F. Eves, Douglas Carroll, Tessa J. Roseboom, Annie T. Ginty, Rebecca C. Painter, Susanne R. de Rooij

**Affiliations:** 1 SpHERE, Faculty of Health Sciences and Sport, University of Stirling, Stirling, FK9 4LA UK; 2 School of Sport, Exercise and Rehabilitation Sciences, University of Birmingham, Birmingham, UK; 3 Centre for Health and Social Care Research, Department of Physical Activity Sciences, Universitat de Vic-Universitat Central de Catalunya, Barcelona, 08500 Vic Spain; 4 Department of Obstetrics & Gynecology, Amsterdam University Medical Centers, University of Amsterdam, Amsterdam, The Netherlands; 5 Department of Epidemiology and Data Science, Amsterdam University Medical Centers, University of Amsterdam, Amsterdam, The Netherlands; 6 Department of Psychology & Neuroscience, Baylor University, TX Waco, USA

**Keywords:** Cohort study, Metabolic health, Public health, Stairs

## Abstract

**Background:**

Stair climbing can be a vigorous lifestyle physical activity, and is associated with healthier lipoprotein profiles, lower body weight and blood pressure, as well as higher aerobic fitness. The present analysis of data from a cohort of late middle-aged men and women examined the association between daily stair climbing and the metabolic syndrome.

**Methods:**

Data from 782 (423 women) participants (mean (SD) age 58.3 (0.95) years in the Dutch Famine Birth Cohort Study (2002–2004) were used to examine the cross-sectional association between self-reported daily stair climbing and the metabolic syndrome. Stair climbing was assessed by the question ‘Do you climb stairs daily?’ and the metabolic syndrome was defined using the established five components relating to lipid fractions, blood glucose levels, blood pressure and abdominal obesity.

**Results:**

Not climbing stairs daily was associated with an increased incidence of the metabolic syndrome (OR = 1.90, 95% CI = 1.23, 2.92, *p* = 0.004) and a greater number of its components (F_1,780_ = 8.48, *p* = 0.004): these associations were still evident after adjusting for a variety of potential confounders.

**Conclusions:**

The most likely explanation for the current findings is that daily stair climbing may be protective against the metabolic syndrome. This result reinforces public health recommendations for increased stair climbing with evidence from physiological outcomes.

## Introduction

New global and national guidance on physical activity suggests that the benefits of physical activity can be accrued across the day [1–3] whereas previously it was thought that activity bouts needed to be of at least 10 min in duration to effect any positive change in health and older guidelines had this recommendation built in [3]. This move towards emphasizing the importance of the accumulation of daily physical activity minutes means that even brief bursts of physical activity, such as stair climbing, may be important in maintaining health and wellbeing. Importantly, such brief bouts of activity as part of everyday life may be more feasible for some individuals than formal exercise sessions. This study reports secondary analyses of data from the Dutch Famine Birth Cohort. The analyses here address a very specific question about brief daily physical activity; is daily stair use protective for cardio-metabolic health?

Climbing stairs can be a vigorous lifestyle physical activity, requiring 9.6 times the energy consumption of sitting for a continuous climb in the field [4] and 8.6 times that of sitting in laboratory conditions when climbing slowly, i.e., 70 steps per minute [5]. The raising of one’s weight against gravity when climbing is an energetically costly behavior which has been objectively characterized as light to vigorous activity depending on intensity and duration; its intensity tends to be underestimated in self-reports [6]. For example, stair climbing and descending at a self-selected pace was reported to be light to vigorous in intensity, and only vigorous during a 10-min but not 1- or 3-min bout. Undoubtedly, inclusion of the lower intensity activity of stair decent in the protocol [4] and testing of a highly fit sample would contribute to subjective reports of light in intensity [7]. Despite this variation in intensity, experimental studies show that increased volumes of stair climbing are associated with improved lipoprotein profiles, reduced weight, blood pressure and fasting blood glucose, as well as increased aerobic fitness and leg strength [7–9]. Additionally, there can be a reduced post-prandial glucose-response to a meal [6]. In observational data, those who report more frequent stair climbing have a decreased risk of coronary heart disease and stroke [8, 10, 11]. Unsurprisingly given its potential benefits, increased volumes of stair climbing are a public health target for many health organizations in the developed world [12–14].

The metabolic syndrome (MetS) is a cluster of symptoms (abdominal adiposity, high triglyceride levels, low levels of high density lipoprotein (HDL) cholesterol, elevated blood pressure, and high levels of fasting blood glucose or diabetes) that increases the risk of cardiovascular disease and all-cause mortality [15, 16]. Although the association between stair climbing and MetS is unknown, the range of beneficial outcomes above certainly suggest that regular stair climbing should be linked to lower risk [9]. Data from the Dutch Famine Birth Cohort allowed us to examine this hypothesis. The paper uses the answer to a single question in the interview about daily stair use to test the utility of the behavior for physiological outcomes.

## Methods

### Participants

In this secondary analysis study, Seven hundred eighty-two Seven participants from the Dutch Famine Birth Cohort (423 women, 359 men), who were born in Amsterdam between November 1943 and February 1947, completed a clinical examination in 2002–4. The primary analyses investigated the effects of reduced energy intake by a mother on the health outcomes for their foetus in adulthood, i.e. prenatal exposure to famine in 1944–1945 [17]. Details are available elsewhere [17, 18], but briefly the cohort were recruited by tracing 5425 people born between November, 1943, and February, 1947 in the Wilhelmina Gasthuis, hospital in Amsterdam, for whom detailed records of the course of gestation and birth from records from the Gemeentearchief (city archive) of Amsterdam were retrieved. The mean caloric rations during the famine were as low as 400–800 cal per day. Following exclusion of those without complete records, this cohort included 2414 singleton liveborns. In 1995, cohort members who were still living in the Netherlands and whose address was known to the investigators were invited to participate in a study on the association between prenatal exposure to the Dutch famine and cardio-metabolic health in later life. In 2002, of the group of 1423 eligible people, 810 agreed to participate, with 782 included in the present analysis having complete data on clinical and stair climbing variables. Fuller details on the whole cohort are published elsewhere [17, 19].

Mean (SD) age at the time of the examination was 58.34 (0.95) years. The study was approved by the appropriate Ethics Committee and conducted in accordance with the declaration of Helsinki. Participants provided informed written consent.

### Measurements

Measurements were taken by trained nurses. Stair climbing was assessed with the simple question ‘Do you climb stairs daily?’ (yes/no). Participants indicated whether they participated in sport for at least an hour each fortnight and their smoking status (current smoker, ex-smoker, never smoker), two lifestyle factors previously associated with MetS risk. For self-reported health, participants rated their health as excellent, very good, good, mediocre or poor [20]. Socio-economic position was measured using the Dutch occupation-based International Socio-economic Index-92 (ISEI-92), a numeric scale based on the person’s or their partner’s occupation, whichever status was higher [21], and marital status was assessed. Participants revealed whether they were taking medication for diabetes and/or hypertension. Triglycerides and HDL were assessed using an enzymatic colorimetric reagent on a P-800 Roche analyzer and plasma glucose levels determined by photometric assay using a Roche Modular P analyzer. Waist circumference was measured by tape midway between the costal margin and the iliac crest. Blood pressure was recorded twice, using an Omron 705 sphygmomanometer and the average value computed. MetS was defined as having at least three of the following: triglyceride levels ≥1.7 mmol/L; HDL < 1.3 mmol/L for women and < 1.03 mmol/L for men; glucose levels ≥5.6 mmol/L and/or taking medication for diabetes; waist circumference ≥ 80 for women and ≥ 94 cm for men; blood pressure ≥ 130/85 mmHg or taking anti-hypertensive medication [22].

The famine period was defined according to the daily official food rations for the general population aged > 21 years. The official rations accurately reflect the variation over time in the total amount of food available in the west of the Netherlands. Foetuses were considered to have been exposed to famine if the average daily rations during any 13-week period of gestation were < 1000 cal, so babies born between 7 January and 8 December 1945 were considered exposed. Periods of 16 weeks each were used to differentiate between people who had been exposed in late gestation (born between 7 January and 28 April 1945), in mid-gestation (born between 29 April and 18 August 1945), and in early gestation (born between 19 August and 8 December 1945). People born before 7 January 1945 and conceived after 8 December 1945 were considered unexposed [19].

### Statistical analysis

Comparisons between stair climbing groups and between those with and without MetS were undertaken using ANOVA and χ^2^. The association between stair climbing and MetS was tested using logistic regression, first in an unadjusted model and then in a model that adjusted for age, sex, socioeconomic position, marital status, smoking, self-reported health and sports participation, and famine exposure; for the last of these, famine exposure in the first trimester was compared to later and no prenatal exposure, as it was for the first trimester that health effects tended to occur [23]. Logistic regression was also used to examine MetS components and daily stair climbing. ANOVA and ANCOVA, using covariates above, tested the association between stair climbing and the number of MetS components.

## Results

The characteristics of those with and without MetS and of those who reported that they did and did not climb stairs daily are presented in Table 1. Those who did not climb stairs daily were at greater risk for MetS in unadjusted (OR = 1.90, 95% CI = 1.23, 2.92, *p* = 0.004), and adjusted models (OR = 1.72, 95% CI = 1.12, 2.64, *p* = 0.01). The multivariate analysis also revealed that MetS was more common in men, in those with poorer self-reported health, and in those who did not participate in sports (see Table 2). The MetS components that contributed most to its association with stair climbing were high blood glucose, (OR = 1.73 95% CI-1.12, 2.66, *p* = 0.01), triglycerides, (OR = 1.49 95% CI = 0.98, 2.28, *p* = 0.07), and blood pressure (OR = 1.50 95% CI = 0.94, 2.39, *p* = 0.09). Not climbing stairs daily was also associated with an increased number of MetS components, in ANOVA, (F_1,780_ = 8.48, *p* = 0.004) and in ANCOVA (F_1,760_ = 6.96, *p* = 0.009). Again, in the multivariate analysis, effects of reporting not climbing stairs survived the addition of the adverse effects of male sex, poor self-reported health, and no sports participation.

**Table 1:** Characteristics of participants with and without MetS and who climb or do not climb stairs every day

Variable	No MetS (*N* = 402)	MetS (*N* = 380)	*p*	Daily Stairs Stairs (*N* = 676)	Not Daily (*N* = 106)	*p*
	Mean (SD) / N (%)			Mean (SD) / N (%)		
Sex (Male)	167 (42)	192 (51)	.01	315 (47)	44 (42)	.33
Age (years)	58.4 (0.96)	58.3 (0.94)	.40	58.3 (0.94)	58.4 (1.03)	.60
**Marital Status**						
Married	303 (76)	294 (78)	.92	527 (78)	70 (66)	.09
Divorced	45 (11)	38 (10)		66 (10)	17 (16)	
Widowed	21 (5)	18 (5)		32 (5)	7 (7)	
Never married	32 (8)	29 (8)		49 (7)	12 (11)	
Socio-economic status	50.2 (14.14)	49.2 (14.23)	.33	50.0 (14.35)	48.2 (13.02)	.22
**Smoking**						
Smoker	98 (24)	94 (25)	.03	156 (23)	36 (34)	.04
Ex-smoker	144 (36)	166 (44)		270 (40)	40 (39)	
Never smoker	160 (40)	119 (31)		249 (37)	30 (27)	
Self-reported health (reverse-scored)	2.8 (0.85)	3.1 (0.88)	<.001	2.9 (0.86)	3.2 (0.95)	.02
Sports participation (Yes)	253 (63)	184 (48)	<.001	380 (56)	57 (54)	.63
Famine exposure in 1st trimester (Yes)	36 (9)	38 (10)	.62	62 (9)	12 (11)	.48
MetS (Yes)	–	–	–	315 (47)	65 (61)	<.01
MetS (N of components)	1.4 (0.71)	3.5 (0.66)	<.001	2.4 (1.27)	2.8 (1.17)	<.01
Daily stair climbing (Yes)	361 (90)	315 (83)	<.01	–	–	–

**Table 2:** Contributors to the presence of MetS in the multivariate analysis

Variable	OR	95% CIs	*p*
Sex (Male)	1.464	1.088–1.969	.01
Age (years)	0.924	0.791–1.079	.32
Marital status	0.916	0.774–1.084	.31
Socio-economic status	1.000	0.989–1.011	.98
Famine exposure, 1st trimester (Yes)	1.114	0.671–1.850	.68
Self-reported health (reverse scored)	1.366	1.444–1.631	<.01
Smoking status	0.938	0.772–1.141	.52
Sport participation (No)	1.580	1.167–1.490	<.01
Daily stairs (No)	1.725	1.117–2.663	.01

## Discussion

Not reporting daily stair climbing was associated with an increased incidence of MetS and possession of an increased number of MetS components. This result is consistent with experimental effects of stair climbing on risk factors for the metabolic syndrome [6, 8, 9, 24] and associations between reported stair use and cardiovascular disease outcomes [8, 10]. Although stair climbing behavior was assessed by self-report, the specificity and simplicity of the posed question was unambiguous; do you climb stairs daily? Responding no to this simple query was associated with increased MetS risk. We can think of no self-presentational reasons for reporting that one does not climb stairs. Finally, avoiding stair climbing on a daily basis can only be achieved by residential circumstance such that one lives on the ground floor, uses no stairs on their commute to work, i.e., chooses the escalator, or selects the lift in their workplace. Observational studies reveal that predominantly, stairs are avoided when there is an alternative means of ascent [25–27] (combined *n* = 1,232,709), more so by older individuals and the female sex, the participants measured in this study [25–27]. The results of the secondary analyses here suggest that ground floor living may be an unhealthy residential choice, and that, where it is within an individual’s control, using stairs in daily commutes or activity and in the workplace would be beneficial for health.

Reverse causation cannot be fully discounted. Individuals in poor health may move from residences with stairs to ground floor accommodation. Set against this interpretation, the association between stair climbing and MetS survived adjustment for self-reported health. Further, reverse causation is rendered less likely given that it was the more unseen components -blood glucose, triglycerides, blood pressure - rather than the more evident one, abdominal adiposity, that appear to be implicated. We adjusted for the potential socio-demographic factors of sex and SES; adjustment for age was inappropriate, given the restricted range in this birth cohort design. Additionally, we adjusted for two behavioral risk factors for MetS, smoking and formal exercise participation, making confounding less likely, although not impossible. The absence of differences between stair climbing sub-groups on sport participation in Table 1 suggests effects of the two physical activity behaviors are independent. However, we acknowledge that sport participation measurement might not have picked up on other confounders, such as the beneficial physical activities of walking or cycling that may occur without being formal exercise. In a recent experimental test, walking up and down stairs at home at a self-selected pace 5 days a week improved the MetS risk factors of body fat, triglycerides, HDL cholesterol and blood glucose [9]. Obviously in residential locations, stair ascent would be followed by a subsequent stair descent which may add to the benefits possible from stair climbing alone [9]. The most plausible explanation for the present results is that daily stair climbing protects against MetS.

It should be acknowledged that a single question, binary subjective measure of stair climbing is a limitation of this research. Although simple, the yes/no question utilized here does not give detail on the frequency of stair climbing each day, the number of flights climbed, and the intensity of climbing. Additionally, a single question cannot assess whether participants maintained this behavior, or any variation in climbing across the week, e.g., such as someone who climbs stairs at work 5 days a week but does not use stairs at the weekend. As noted above, however, stair climbing is typically avoided at work [26], with avoidance more frequent in overweight individuals that would be more likely to have MetS. Detailed interviewing would have allowed for much more nuanced analyses in terms of stair climbing dosage for effects on health. The objective observation of stair climbing utilized in many quasi-experimental designs e.g. [27],, naturally would remove the doubts about reliability inherent in all subjective self-report measures, and specifically the under-estimation of intensity that may be occurring in stair climbing self-reports [24]. That being said, that we still find an association even with this simple measure suggests that the impact of stair climbing is considerable, and opens up the field for further research into aspects such as dose-response relationships. This is supported by recent research showing that even subjectively-reported ‘light’ and short bouts of stair climbing can affect the metabolic response to a meal [24], and this response is not influenced by underlying cardio-respiratory fitness [28]. This also draws attention to the possible attenuation of the post-prandial glucose peak which may be one plausible mechanism by which stair climbing helps reduce the risk of MetS.

Although the current sample is somewhat unusual in its recruitment regarding the Dutch famine exposure, we believe it is unlikely that the famine exposure element of this cohort limits the generalizability of the present results given that not all of the sample were famine exposed, and famine exposure did not significantly influence metabolic syndrome in this sample [17]. Further, we do not believe that the age of these data affects the current relevance of these findings, as both sedentary behavior and the metabolic syndrome are common, if not increasingly so today. However, these results could be regarded as preliminary until further longitudinal research is able to confirm these associations.

Encouragingly, stair climbing interventions are one of the few physical activity initiatives that repeatedly change behavior [29, 30]. It has been estimated that the increased energy consumption from these interventions is six times more cost-effective than their nearest competitor [31]. There is, however, a practical limitation to stair climbing as a plausible approach to increasing public health. The relatively brief duration of a single stair climbing episode in public access settings means that the behavior might have minimal effects on overall accumulation of physical activity during daily life [26, 31, 32]. Instead, repeated stair climbing at work, or in the home environment as reported here, would allow daily accumulation of stair climbing episodes [26, 32] . Unlike formal exercise sessions such as sport, stair climbing is a plausible behavior for most of the population. No particular skills are required, there is no competition, and there are few presentational concerns. Importantly, there are no time barriers to stair climbing, a frequent reason given for insufficient participation in other types of physical activity. Indeed, greater effects of stair climbing interventions have been reported in the overweight, suggesting that it is a physical activity that is acceptable to those at greater risk of MetS [32, 33]. Finally, research suggests that it is the intensity of physical activity, rather than its duration, that may be more important for MetS [34–36]. In this respect, the vigorous nature of stair climbing may be a particularly beneficial property of this lifestyle activity, particularly given that individuals tend to underestimate how intense stair climbing may actually be [6]. Stair climbing is promoted for its potential health benefits, despite relatively limited evidence of efficacy on health outcomes. Here, we report associations between daily stair use and physiological markers of cardio-metabolic health.

## Data Availability

The Dutch famine birth cohort study welcomes opportunities for collaboration; enquiries should be directed to the principal investigator (t.j.roseboom@amsterdamumc.nl).
